# Iron Exposure and the Cellular Mechanisms Linked to Neuron Degeneration in Adult Mice

**DOI:** 10.3390/cells8020198

**Published:** 2019-02-24

**Authors:** Lin-Bo Li, Rui Chai, Shuai Zhang, Shuang-Feng Xu, Yan-Hui Zhang, Hai-Long Li, Yong-Gang Fan, Chuang Guo

**Affiliations:** College of Life and Health Sciences, Northeastern University, Shenyang 110819, China; 18640176710@163.com (L.-B.L.); m1537307231@163.com (R.C.); icnmif@126.com (S.Z.); 13804021867@163.com (S.-F.X.); zhangyanhui@cmu.edu.cn (Y.-H.Z.); LiHailong1166@gmail.com (H.-L.L.); 18841609869@163.com (Y.-G.F.)

**Keywords:** Alzheimer’s disease, iron, neuron loss, apoptosis, autophagy, ferroptosis

## Abstract

Although the causal relationship between Alzheimer’s disease (AD) and iron overload remains unclear, iron dyshomeostasis or improper transport mechanisms are speculated to lead to the accumulation of this neurotoxic metal in the hippocampal formation and other cerebral areas related to neurodegenerative diseases, resulting in the formation of reactive oxygen species (ROS) and, ultimately, cell death. In this study, exposure to high dietary iron (HDI) revealed no significant difference in the number of iron-positive cells and iron content in the cortex and hippocampal region between wild-type (WT) and *APP/PS1* mice; however, compared with the control mice, the HDI-treated mice exhibited upregulated divalent metal transporter 1 (DMT1) and ferroportin (Fpn) expression, and downregulated transferrin receptor (TFR) expression. Importantly, we confirmed that there were significantly fewer NeuN-positive neurons in both *APP/PS1* and WT mice given HDI, than in the respective controls. Moreover, this iron-induced neuron loss may involve increased ROS and oxidative mitochondria dysfunction, decreased DNA repair, and exacerbated apoptosis and autophagy. Although HDI administration might trigger protective antioxidant, anti-apoptosis, and autophagy signaling, especially in pathological conditions, these data clearly indicate that chronic iron exposure results in neuronal loss due to apoptosis, autophagy, and ferroptosis, hence increasing the risk for developing AD.

## 1. Introduction

Alzheimer’s disease (AD) is the most common neurodegenerative disease, and is characterized by senile plaques (SPs) formed from β-amyloid (Aβ) [1] and neurofibrillary tangles (NFTs) composed of tau protein [2]. Both SPs and NFTs decrease synaptic plasticity and neuronal connectivity and lead to neuron dysfunction and death, ultimately resulting in cognitive damage and dementia [3].

Studies have implicated cellular overload of iron, a necessary cofactor in many metabolic processes in the central nervous system, along with iron-induced oxidative stress, in the development of AD [4,5,6]. Excessive iron levels may lead to the dissociation of iron regulatory proteins (IRPs), which function as cytosolic iron sensors, from their iron responsive element (IRE) binding sites and abolish their repression of APP mRNA, resulting in enhanced APP translation [7]. In contrast, the translation of APP mRNA and Aβ generation can be inhibited by chemical IRE inhibitors [7]. Furthermore, iron is able to bind to Aβ, induce Aβ aggregation, accelerate the formation of oligomers, and enhance the toxicity of Aβ [8]. Interestingly, there is some evidence that APP aids iron export from neurons through its interaction with ferroportin (Fpn), and APP knockout mice reveal markedly increased hippocampal and cortical neuronal iron and oxidation [9]. Emerging evidence suggests that sAPPα, a cleavage product of the non-amyloidogenic pathway, stabilizes Fpn in the neuronal membrane, supporting the neuroprotective properties of APP [9,10].

In addition to Aβ, iron has been found to be relevant to the formation of NFTs in AD patient brains [11,12]. Furthermore, iron has the ability to bind to hyperphosphorylated tau and cause tau aggregation in vitro [13]. In an in vivo mouse model of AD, iron exposure induced tau hyperphosphorylation [14,15], which can occur under conditions of increased oxidative stress [16,17]. On a similar note, another study suggested that the loss of soluble tau may contribute to iron retention in the brain in tau-knockout mice and in primary cultured neurons [18]. Furthermore, iron levels have been verified to be significantly higher in the brains of P301S transgenic mice, which carry the human Tau gene with the P301S mutation, than in the brains of wild-type (WT) mice, whereas iron chelation treatment with α-lipoic acid (LA) (a naturally occurring enzyme cofactor with antioxidant and iron chelator properties) has neuroprotective potential due to mitigating oxidative stress, inflammation, neuronal degeneration, and tauopathy, through the redistribution of iron in specific areas of the mouse brain associated with neurodegeneration [19]. Based on these findings, whether excess iron exposure plays a mechanistic role in amyloid and/or tau pathology, and whether APP and/or tau pathology disrupt local iron homeostasis, remain unclear.

Of course, pathological neuronal iron accumulation has been observed in AD brains [9,20]. Increasing evidence suggests that SPs and NFTs are involved in neurotoxicity [21], apoptosis [22], autophagy [23], ferroptosis [24], oxidative stress [25], and DNA damage [26] during AD development and progression. Further support for the interlinkage between iron metabolism and AD comes from the observations that therapeutic strategies using iron chelators and/or IRE inhibitors may attenuate SPs and NFTs, as well as prevent neuron loss, in various in vivo AD models [7,27]. As such, the types of cell death that are responsible for the bulk of neuronal loss in AD in the absence or presence of excess iron remains unclear. Here, we hypothesized that excess iron exposure may exaggerate age-dependent, amyloid-mediated neurotoxicity and neuronal dysfunction. Therefore, we sought to determine the molecular mechanism responsible for this dysfunction using APPSwe/PS1DE9 (*APP/PS1*) mice and littermate C57BL/6J mice.

In this study, we evaluated the forms of neuron death and their mechanisms in WT mice and the *APP/PS1* double Tg mouse model of AD after treatment with high iron in the drinking water. We detected factors related to neurotoxicity, apoptosis, autophagy, ferroptosis, oxidative stress, and DNA damage. Our results showed that a high level of iron-induced neuron death is caused by a mixture of factors in both the normal and pathological conditions.

## 2. Materials and Methods

### 2.1. Animals and Treatment

*APP/PS1 (APPswe/PSEN1dE9)* double Tg mice and C57BL/6J (WT) mice were originally obtained from Jackson Laboratory (West Grove, PA, USA). The mice were maintained in a controlled environment (22–25 °C, 40–60% relative humidity, and 12 h light/dark cycle), with a standard diet and distilled water available ad libitum. For subsequent experiments, we intercrossed these mice to generate *APP/PS1* and WT littermate mice. All experimental procedures using animals were designed to minimize suffering and the number of subjects used. These studies were conducted in accordance with the guidelines for the care and use of medical animals developed by the Ministry of Health of the People’s Republic of China (1998), and the ethical standards for laboratory animals in Northeastern University (#161031).

We divided the 9-month-old male mice into four groups: C57BL/6J (WT), C57BL/6J + Fe (WT + Fe), *APP/PS1* (AD) and *APP/PS1* + Fe (AD + Fe) (*n* = 8 in each group). The high-iron groups were treated with 5 g/L ferric ammonium citrate (FAC) (Sinopharm Chemical Reagent Co., Ltd., Beijing, China) for three months, and the control groups (C57BL/6J and *APP/PS1*) were given distilled water.

### 2.2. Tissue Preparation

Three months later, control- and FAC-treated mice were anesthetized with sodium pentobarbital (50 mg/kg, intraperitoneally) and then transcardially perfused with 0.9% saline. After the blood was completely perfused, the mouse brain was then immediately removed and cut in half on an icy plate along the sagittal plane. One half of the brain was retained in 4% paraformaldehyde for morphological evaluation, and the other half was frozen at −80 °C for the molecular biology experiments.

### 2.3. Perl’s-Diaminobenzidine (DAB) Iron Staining

The frozen brain sections were hydrated with distilled water for 5 min. Then, a mixture of 2% K_4_ [Fe (CN)_6_] (Sinopharm Chemical Reagent Co., Ltd., Beijing, China) and 2% HCl was dropped on sections, followed by incubation at room temperature for 30 min. After being washed three times by 0.01 M PBS, the sections were stained with diaminobenzidine (DAB) dye solution (30 mg DAB (Beijing Labeled Biotech CO., LTD, Beijing, China) + 200 mL TrisHCL + 60 μL 30% H_2_O_2_) for 3 min, with observation under an optical microscope. The microscopic brown particles were considered positive staining. Images of stained sections were collected on a light microscope (DM4000B; Leica, Wetzlar, Germany). After three repeated experiments, the images were quantified using ImageJ analysis software (Version 4.0, National Institutes of Health, Rockville, MA, USA), and the area of positive staining was counted. Then, Graphpad prism software (Version 5.0, Graphpad, La Jolla, CA, USA) was used for differential analysis. Subsequent staining results were analyzed using the same method.

### 2.4. Atomic Absorption Spectroscopy (AAS)

Brain tissue samples were accurately weighed with an analytical balance, and the samples were treated with 200 μL concentrated nitric acid at 98 °C for 15 min. After cooling to room temperature, the samples were diluted to 5 mL with 1% nitric acid. A graphite furnace atomic absorption spectrometer (ZEEnit700P, Analytikjena, Jena, Germany) was used to detect the iron level.

### 2.5. Immunohistochemistry (IHC)

The mouse brain was removed from 4% paraformaldehyde, dehydrated, embedded in paraffin, and finally cut into 5 μm sections. The coronal paraffin sections were then dewaxed and rehydrated, and treated with endogenous peroxidase inhibitor (MXB Biotechnologies, Fuzhou, China) for 10 min. L.A.B solution (Polyscience, Inc., Niles, IL, USA) was added for 20 min to retrieve the antigens. After each treatment, the sections were washed three times with 0.01 M PBS for 5 min each time, and blocked with goat serum for 30 min. Then, the sections were incubated with only one primary antibody overnight at 4 °C. The primary antibodies used for immunohistochemistry were rabbit anti-GFAP (1:400, Sigma-Aldrich, Philadelphia, PA, USA) and rabbit anti-ionized calcium-binding adapter molecule 1 (Iba1) (1:400, Cell Signaling Technology, Danvers, MA, USA). Next, the sections were treated with the corresponding biotinylated secondary antibody (MXB Biotechnologies, Fuzhou, China) for 1 h at room temperature, and then treated with HRP-streptavidin for 30 min. The staining steps were identical to Perl’s-DAB iron staining. The samples were then immersed in distilled water to stop the reaction, and finally counterstained with hematoxylin. After these steps were completed, the sections were dehydrated and sealed. Images of the stained sections were collected using an optical microscope (DM4000B; Leica, Wetzlar, Germany).

### 2.6. Immunofluorescence (IF)

The frozen brain sections were rehydrated and treated with L.A.B. solution (Polyscience, Inc., Niles, IL, USA) for 20 min. They were then blocked with goat serum for 30 min, and incubated overnight at 4 °C with both the mouse-anti-Aβ (1:400) and rabbit-anti-NeuN (1:400, Cell Signaling Technology, Danvers, MA, USA) antibodies. Subsequently, sections were incubated in DyLight 594-labeled goat anti-mouse IgG and 488-labeled goat anti-rabbit IgG (1:400, Cell Signaling Technology, Danvers, MA, America) at room temperature for 1 h. Finally, the sections were labeled with 4′,6-diamidino-2-phenylindole (DAPI) (Beyotime Institute of Biotechnology, Beijing, China) and sealed with anti-fluorescence quencher (Beyotime Institute of Biotechnology, Beijing, China). The sections were observed and photographed using a confocal fluorescence microscope (Leica, SP8., Wetzlar, Germany).

### 2.7. Nissl Staining

The frozen brain sections were hydrated with distilled water for 5 min, incubated in Nissl staining solution (Beyotime Institute of Biotechnology, Beijing, China) for 10 min at room temperature, washed twice with distilled water for a few seconds, and placed into 95% ethanol for 5 min, followed by 100% ethanol for 2 min. The sections were then cleared with xylene for 10 min and sealed. The images of the stained sections were collected with a light microscope (DM4000B; Leica, Wetzlar, Germany).

### 2.8. Western Blotting

Mouse brain homogenates were lysed in RIPA buffer (Beyotime Institute of Biotechnology, Beijing, China) containing protease inhibitor cocktail, and then centrifuged at 13,000 r/min for 25 min at 4 °C. The supernatants were collected, and the total protein levels were measured using an ultraviolet (UV) 1700 PharmaSpec ultraviolet spectrophotometer (Shimadzu, Tokyo, Japan). Thirty micrograms of protein were loaded onto 10% SDS polyacrylamide gels, and transferred onto polyvinylidene fluoride (PVDF) membranes (Millipore, Billerica, MA, USA). After the membranes were blocked with 5% nonfat milk in TBS containing 0.1% Tween-20 for 1 h, the transferred PVDF membranes were probed overnight with the following antibodies: goat anti-AIF (1:2000; Santa Cruz Biotechnology, Dallas, TX, America), rabbit anti-Bax (1:1000; Santa Cruz Biotechnology), mouse anti-Bcl-2 (1:1000; Santa Cruz Biotechnology), mouse anti-Beclin-1(1:3000; Santa Cruz Biotechnology), rabbit anti-caspase 3 (1:1000; Cell Signaling Technology), rabbit anti-DMT1 (1:1000; Cell Signaling Technology), rabbit anti-Fpn (1:1000; Cell Signaling Technology), mouse anti-GFAP (1:1000; Santa Cruz Biotechnology), rabbit anti-GPX4 (1:1000; Santa Cruz Biotechnology), rabbit anti-Iba1 (1:1000; Abcam), rabbit anti-LC3 A/B (1:3000; Cell Signaling Technology), rabbit anti-MTH1 (1:1000; Santa Cruz Biotechnology), rabbit anti-m-TOR (1:1000; Cell Signaling Technology), mouse anti-MUTYH (1:3000; Santa Cruz Biotechnology), mouse anti-OGG1 (1:2000; Santa Cruz Biotechnology), rabbit anti-P62 (1:1000; Cell Signaling Technology), rabbit anti-PARP1 (1:1000; Santa Cruz Biotechnology), rabbit anti-p-m-TOR (1:1000; Cell Signaling Technology), rabbit anti-SOD1 (1:1000; Cell Signaling Technology), mouse anti-TFR(1:1000; Cell Signaling Technology), rabbit anti-xCT (1:1000; Abcam, Cambridge, UK), and mouse anti-β-actin (1:10000; Cell Signaling Technology). The immunoblots were washed and treated with the appropriate species of horseradish peroxidase (HRP)-conjugated secondary antibody (1:10000; Beijing Zhongshan Jinqiao Biotechnology Co., Ltd., Beijing, China), and immunological complexes were visualized by enhanced chemiluminescence (ECL) kits (Tanon, Shanghai, China) using the ChemiDoc XRS system and the accompanying Quantity One software (Bio-Rad, Hercules, CA, USA). The immunoreactive bands were quantified using ImageJ analysis software.

### 2.9. Detection of Malondialdehyde (MDA) and Reactive Oxygen Species (ROS)

The brain tissue was placed in 0.1 M PBS solution at a 1:9 ratio of brain tissue (g) to PBS volume (mL), ultrasonically fragmented, and centrifuged at 1000 r/min for 10 min. The supernatant was removed, and the protein concentration was determined. The relevant indicators were then measured using commercial assay kits (Jiancheng Biochemical, Nanjing, China). In brief, the ROS levels were measured by adding 5 μL of supernatant and 195 μL of 2′,7′-dichlorofluorescein diacetate (DCFH-DA) (Jiancheng Bioengineering Institute, Nanjing, China) to a 96-well plate, which was incubated for 30 min at 37 °C in the dark. Fluorescence detection was then carried out using an excitation wavelength of 500 nm and an emission wavelength of 525 nm (Synergy/H1, BioTek, Jiangsu, China). The detection result was expressed by the fluorescence value/mg protein. Malondialdehyde (MDA) levels were assessed following the manufacturer’s instructions, and the samples were incubated at 95 °C for 40 min. The absorbance was measured at 532 nm to calculate the MDA levels, which were expressed as nmol per mL. Protein concentrations were measured by a BCA protein assay (Beyotime Biotechnology, Beijing, China).

### 2.10. Statistical Analysis

All results were obtained from three repeated experiments and presented as the mean ± SEM. The difference between groups was analyzed by the unpaired two-tailed Student t test for the two data sets, or the one-way analysis of variance (ANOVA) for the four data sets. The results were reported to be highly statistically significant if *p* < 0.01, and statistically significant if *p* < 0.05.

## 3. Results

### 3.1. Effect of High Dietary Iron (HDI) on Iron and Iron-Transport-Related Proteins in the Wild-Type (WT) and APP/PS1 Mouse Brain

**Figure 1:**
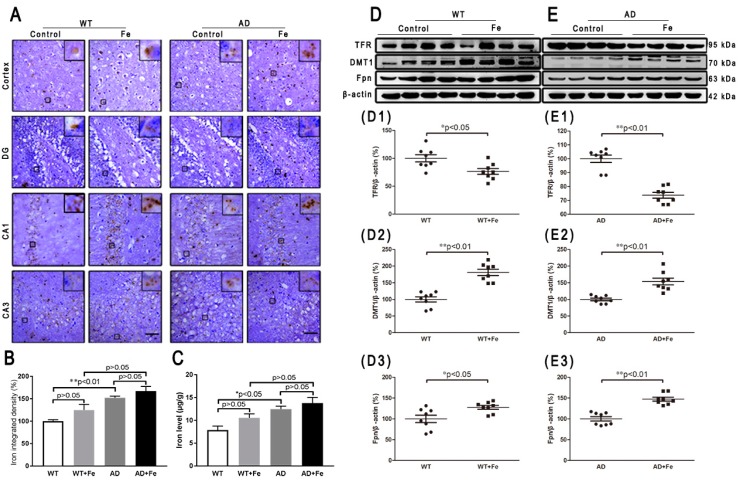
Effect of high dietary iron on iron and iron-transport-related proteins in the mouse brain. (**A**) Perl’s diaminobenzidine (DAB) iron staining showed that high dietary iron (HDI) could increase the number of iron-positive cells in the cortex and hippocampal region of wild-type (WT) and *APP/PS1* mice. (**B**) Quantitative analyses of Perl’s-DAB iron staining. Scale bar = 50 μm. (**C**) The results of iron atomic absorption spectroscopy (AAS). (**D**, **E**) Western blot analysis of transferrin receptor (TFR), divalent metal transporter 1 (DMT1) and ferroportin (Fpn). (**D1**–**D3**, **E1**–**E3**) Quantitative analyses of Western blot for TFR, DMT1 and Fpn. β-actin was used as an internal control. All results are presented as the mean ± standard error of the mean (SEM) (*n* = 8). * *p* < 0.05, ** *p* < 0.01 compared with the control group.

To investigate the reason for the HDI-induced neurodegeneration in the mouse brain, we first examined the level of iron and iron-related transporter proteins. Perl’s-DAB iron staining showed that HDI increased the number of iron-positive cells in the cortex and hippocampal region in WT and *APP/PS1* mice (Figure 1A), however the increase was not statistically significant in the brains of either WT or *APP/PS1* mice after treatment with HDI (Figure 1B, *p* > 0.05). Simultaneously, we used AAS to evaluate the iron content (Figure 1C), and the results suggested that iron levels were significantly higher in the brains of *APP/PS1* mice than in WT mice, but HDI did not statistically alter the iron content in the brains of either WT or *APP/PS1* mice. Next, we examined the effect of HDI on the expression of transferrin receptor (TFR), divalent metal transporter 1 (DMT1), and Fpn—the only iron export protein of neurons (Figure 1D,E). TFR expression in the brain was significantly decreased after HDI treatment in both WT and *APP/PS1* mice (Figure 1(D1,E1), *p* < 0.05 or *p* < 0.01, respectively). Nevertheless, the expression of DMT1 and Fpn was significantly increased after HDI treatment in both the WT and *APP/PS1* mouse brains (Figure 1(D2,E2,D3,E3), *p* < 0.05 or *p* < 0.01, respectively). These results suggested that exogenous iron might penetrate the blood–brain barrier (BBB) and enter into the central nervous system (CNS) of adult mice, to induce iron redistribution by regulating the expression and function of brain iron-transport-related proteins.

### 3.2. Effect of HDI on Neurodegeneration in the WT and APP/PS1 Mouse Brain

**Figure 2:**
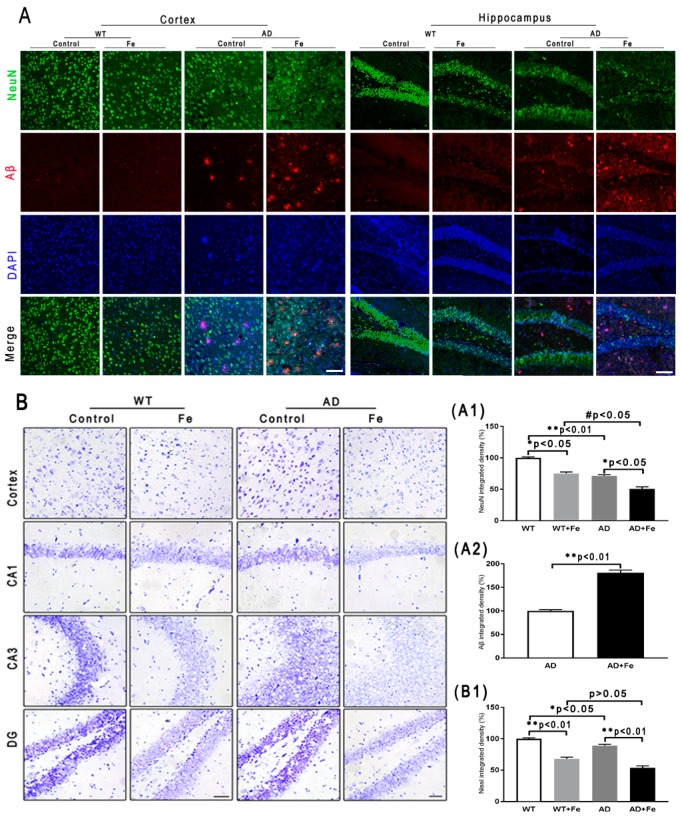
(**A**) Immunofluorescence of Aβ and NeuN. Scale bar = 25 μm, (**A1**,**A2**) Quantitative analyses of Neun-positive staining and Aβ-positive staining. (**B**) Nissl staining in the mouse brain. Scale bar = 25 μm. (**B1**) Quantitative analyses of Nissl-positive staining, *n* = 8. * *p* < 0.05, ** *p* < 0.01 compared with the control group; # *p* < 0.05 compared with the iron-treated WT group.

**Figure 3:**
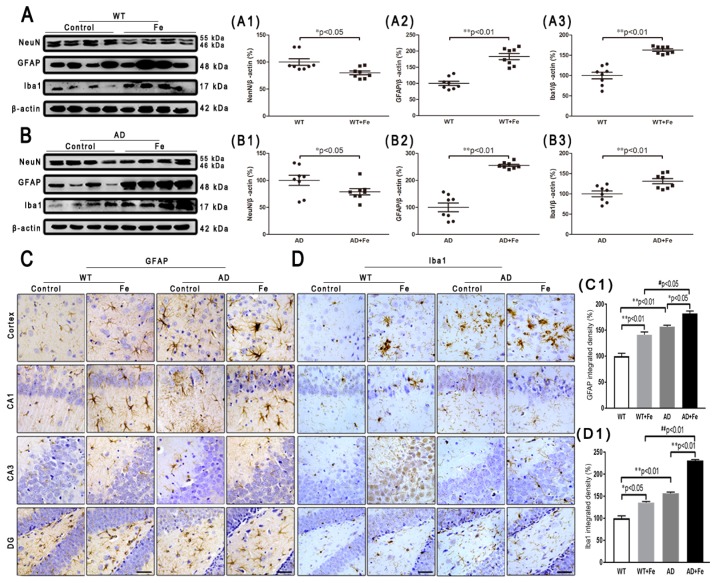
Effect of HDI on neurons and glia. (**A**, **B**) Western blot analysis of NeuN, glial fibrillary acidic protein (GFAP) and ionized calcium-binding adapter molecule 1 (Iba1). (**A1**–**A3**, **B1**–**B3**) Quantitative analyses of Western blot for NeuN, GFAP and Iba1. β-actin was used as an internal control. (**C**, **D**) Immunohistochemistry staining of GFAP and Iba1. (**C1**, **D1**) Quantitative analyses of GFAP-positive astrocytes staining and Iba1-positive microglia staining. Scale bar = 50 μm. All results are presented as the mean ± SEM (*n* = 8). * *p* < 0.05, ** *p* < 0.01 compared with the control group; # *p* < 0.05, # # *p* < 0.01 compared with the iron-treated WT group.

To characterize the effects of HDI on the neuropathology of AD, we assessed changes in Aβ plaques, neurons, glial cells, and associated marker proteins in the brains of *APP/PS1* mice and WT littermate controls at 12 months of age. As shown in Figure 2A, immunofluorescence demonstrated that HDI obviously decreased the fluorescence intensity of NeuN-positive neurons in the cortex and hippocampus of the WT and *APP/PS1* mouse brains, relative to their respective controls (Figure 2(A1), *p* < 0.05 or *p* < 0.01, respectively). Meanwhile, the NeuN-positive neurons in the cortex and hippocampus of HDI-treated *APP/PS1* mice were apparently reduced compared with HDI-treated WT mice, accompanied by an increase of Aβ-positive plaques (Figure 2(A1,A2), *p* < 0.05 or *p* < 0.01, respectively). We then aimed to define the functional state of these neurons in the cortex and hippocampus by Nissl staining. In both tissues, Nissl body levels were decreased by HDI treatment in both *APP/PS1* mice and WT littermate controls (Figure 2(B,B1), *p* < 0.05 or *p* < 0.01, respectively), suggesting that HDI treatment inhibits the activity of neurons. We also examined NeuN protein expression in the brain tissues of HDI-treated *APP/PS1* mice and WT mice, and found that NeuN protein expression was markedly downregulated compared to that in the respective controls (Figure 3(A1,B1), *p* < 0.05 or *p* < 0.01, respectively). This is consistent with the idea that chronic excess iron exposure might significantly undermine the structure and functions of specific neurons, ultimately causing neuronal loss.

As with neurons, glial cells are also involved in the regulation of iron homeostasis in the brain [28]. The Western blot results (Figure 3A,B) revealed a significant increase in the expression level of glial fibrillary acidic protein (GFAP) and ionized calcium-binding adapter molecule 1 (Iba1) in the WT and *APP/PS1* mouse brains after HDI (Figure 3(A2,A3,B2,B3), *p* < 0.05 or *p* < 0.01, respectively). Simultaneously, in both the cortex and hippocampus of HDI-treated WT and *APP/PS1* mice, immunohistochemistry staining demonstrated a sharp increase in GFAP-positive astrocytes and Iba1-positive microglia, with thick processes and enlarged somata (Figure 3(C,D,C1,D1), *p* < 0.05 or *p* < 0.01, respectively), indicating activation of astrocytes and microglia, respectively. Although previous morphological analysis has indicated that AD tissue is associated with activated microglia and Aβ plaques [29,30], whether the cells lacking Aβ plaques are microglia is not readily apparent.

These results indicated that HDI could distinctly activate glial cells and increase Aβ-induced neuroinflammation in the *APP/PS1* mouse brain. 

### 3.3. HDI-Induced Redox Status and DNA Damage Depends Upon Aβ Status in the Mouse Brain

**Figure 4:**
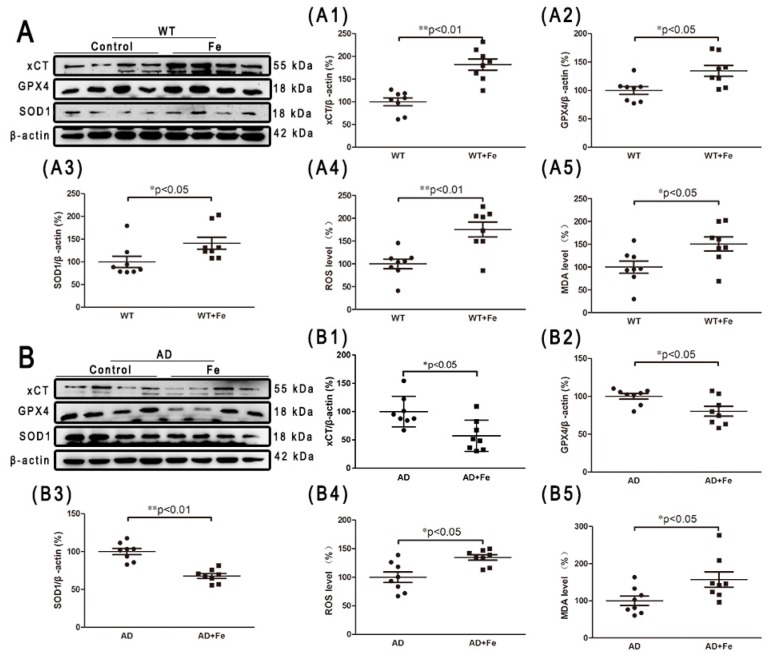
HDI-induced redox status in the mouse brain. (**A**, **B**) Western blot analysis of glutamate-cystine transporter (xCT), glutathione peroxidase 4 (GPX4), and superoxide dismutase (SOD1). (**A1**–**A3**, **B1**–**B3**) Quantitative analyses of Western blot for xCT, GPX4, and SOD1. β-actin was used as an internal control. (**A4**, **A5**, **B4**, **B5**) Quantitative analyses of reactive oxygen species (ROS) and malondialdehyde (MDA). All results are presented as the mean ± SEM (*n* = 8). * *p* < 0.05, ** *p* < 0.01.

Iron is potentially toxic due to the generation of free radicals and ROS through the Fenton reaction. However, the human body is equipped with detoxifying mechanisms that alter redox regulation, and can even repair damage caused by reactive species [31]. To examine whether similar metabolic changes occur in HDI-treated WT and *APP/PS1* mouse brains, we performed a series of experiments to detect the oxidative stress index and endogenous antioxidants. As shown in Figure 4, chronic iron exposure was associated with enhanced ROS and MDA, suggesting that HDI increased the level of free radicals and induced and aggravated oxidative stress in both the WT and *APP/PS1* mouse brains, especially in the former. Glutamate-cystine transporter (xCT) is a cystine-glutamate antiporter. It transports extracellular cystine into cells, which is converted to cysteine for glutathione (GSH) synthesis [32]. Glutathione peroxidase 4 (GPX4) is an intracellular antioxidant enzyme that reduces the production of phospholipid peroxide in cell membranes [32]. Superoxide dismutase (SOD1) is a Cu/Zn superoxide dismutase, a major antioxidant enzyme that catalyzes the conversion of superoxide into hydrogen peroxide and molecular oxygen. In this study, HDI-treated WT mice showed significantly higher expression levels of xCT, GPX4, and SOD1 than the nontreated controls (Figure 4(A,A1–A5), *p* < 0.05 or *p* < 0.01, respectively). Conversely, the expression levels of these antioxidants were significantly decreased in the *APP/PS1* mouse brains after HDI treatment (Figure 4(B,B1–B5), *p* < 0.05 or *p* < 0.01, respectively). These results suggested that the brain may trigger a compensatory response to iron-induced oxidative damage under normal physiological conditions, while the compensatory response is severely inhibited in conditions of Aβ accumulation.

**Figure 5:**
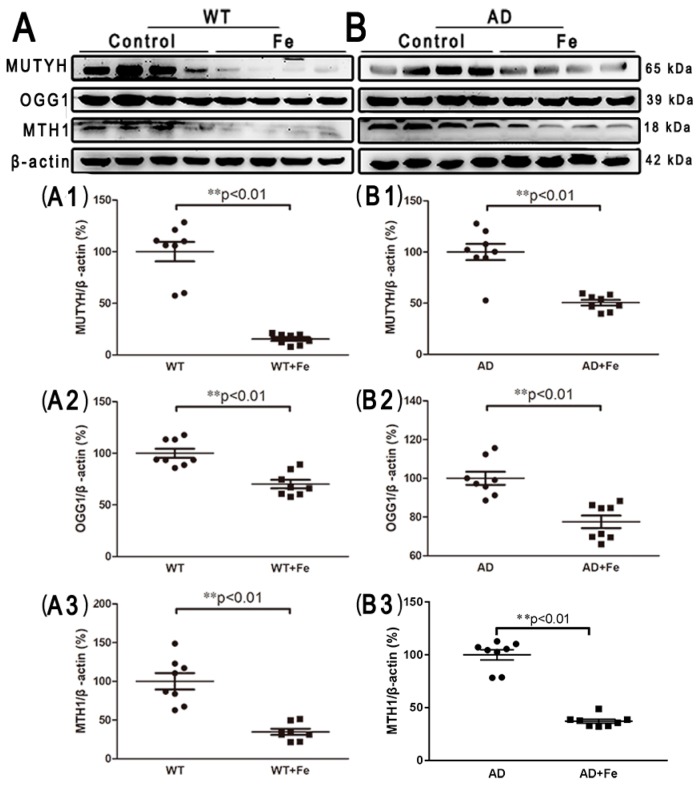
HDI-induced oxidative DNA damage in the mouse brain. (**A**, **B**) Western blot analysis of MutY homologous DNA glycosidase (MUTYH), 8-hydroxyguanine DNA glycosidase (OGG1), and 8-hydroxy guanine nucleotidase (MTH1). (**A1**–**A3**, **B1**–**B3**) Quantitative analyses of Western blot for MUTYH, OGG1, and MTH1. β-actin was used as an internal control. All results are presented as the mean ± SEM (*n* = 8). ** *p* < 0.01.

Additionally, in order to detect iron-induced oxidative DNA damage, several DNA damage repair proteins, including mammalian MutY homologous DNA glycosidase (MUTYH), 8-hydroxyguanine DNA glycosidase (OGG1) and 8-hydroxy guanine nucleotidase (MTH1), were employed. After HDI treatment, the expression levels of MUTYH, OGG1, and MTH1 were significantly decreased in both the WT (Figure 5(C,C1–C3), *p* < 0.01) and *APP/PS1* (Figure 5(D,D1–D3), *p* < 0.01) mouse brains, suggesting that HDI may induce DNA damage, resulting in downstream events leading to neuron loss.

### 3.4. Changes in Apoptosis and Autophagy-Related Proteins in the Brains of HDI-Treated Mice

**Figure 6:**
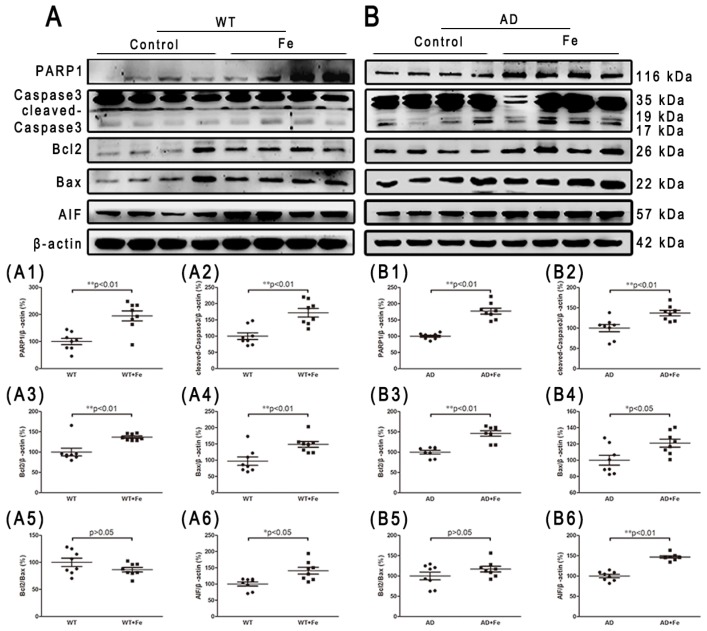
Changes in apoptosis-related proteins in the brains of HDI-treated mice. (**A**, **B**) Western blot analysis of PARP1, caspase 3, Bcl2, Bax, and AIF. (**A1**–**A6**, **B1**–**B6**) Quantitative analyses of Western blot for PARP1, caspase 3, Bcl2, Bax and AIF. β-actin was used as an internal control. All results are presented as the mean ± SEM (*n* = 8). * *p* < 0.05, ** *p* < 0.01.

Iron overload and oxidative damage are known to drive neuronal cell death [33,34]. Therefore, we determined whether *APP/PS1* mice developed the same rate of neuron loss as WT mice, with or without HDI. Apoptosis and autophagy are the main causes of neuron loss in AD-affected brains [35,36,37,38]. In HDI-treated WT mice, HDI significantly induced apoptosis through an increase in the expression of PARP1, cleaved-caspase 3 and AIF, but the Bcl-2/Bax ratio in the brain was not significantly different between HDI-treated and untreated WT mice (Figure 6(A,A1–A6), *p* < 0.05 or *p* < 0.01, respectively). Similar changes in apoptosis-associated proteins were seen in the brains of HDI-treated AD mice relative to their respective controls, although these changes were weaker (Figure 6(B,B1–B6), *p* < 0.05 or *p* < 0.01, respectively).

**Figure 7:**
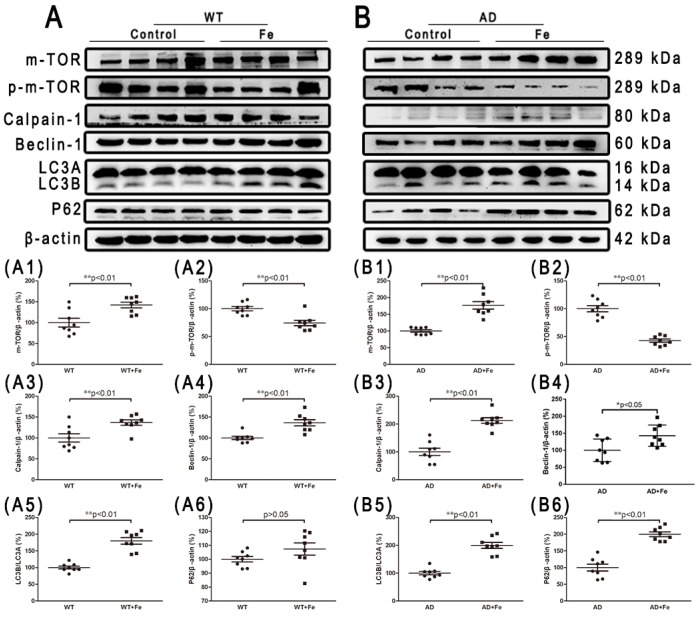
Changes in autophagy-related proteins in the brains of HDI-treated mice. (**A**, **B**) Western blot analysis of m-TOR, p-m-TOR, calpain 1, beclin 1, LC3 A/B, and P62. (**A1**–**A6**, **B1**–**B6**) Quantitative analyses of Western blot for m-TOR, p-m-TOR, calpain 1, beclin 1, LC3 A/B, and P62. β-actin was used as an internal control. All results are presented as the mean ± SEM (*n* = 8). * *p* < 0.05, ** *p* < 0.01.

Given the prominent role of chronic iron exposure in AD, and the increased autophagy latency in the development of severe neuron loss caused by iron accumulation, we then investigated the contribution of autophagy to brain damage. m-TOR is an evolutionarily conserved PI3-kinase family member that plays a key role in integrating different biochemical and growth factor signals, and regulates the occurrence of autophagy [39]. We found that HDI treatment did not significantly affect m-TOR expression in WT mice, but significantly increased its expression in AD model mice (Figure 7(A1,B1), *p* < 0.01), whereas HDI treatment significantly decreased p-m-TOR expression in both groups (Figure 7(A2,B2), *p* < 0.01). Calpain, which is independent of m-TOR, participates in intracellular autophagy and apoptosis by cleaving various protein substrates [40]. Here, calpain-1 protein expression was significantly higher in the brains of HDI-treated WT and *APP/PS1* mice than in the brains of the respective controls (Figure 7(A3,B3), *p* < 0.01). Next, the protein levels of Beclin-1, LC3A/B, and p62, which are reliable markers of autophagy [41], were probed. We found that the level of p62 was dramatically elevated by HDI treatment in the brains of *APP/PS1* mice (Figure 7(B6), *p* < 0.01), whereas the levels of Beclin-1 and LC3A/B were significantly increased in the brains of both WT and *APP/PS1* mice after HDI treatment (Figure 7(A4,A5,B4,B5), *p* < 0.05 or *p* < 0.01, respectively).

Taken together, our results suggest that excessive iron exposure plays a critical role in regulating neuron loss.

## 4. Discussion

Despite the evidence regarding the pathological accumulation of iron in AD brains [42,43], only a few studies have demonstrated that iron exposure can result in iron entering the brain, leading to possible protein aggregation and neuron vulnerability. In the present study, we systematically showed that chronic exposure to iron for mice caused a disorder of membrane-transport protein function and intracellular iron homeostasis, and resulted in a significant increase in ROS and free radical MDA, ultimately leading to neuron and glial cell dysfunction and even neuron loss.

Experimental iron overload using dietary supplementation with iron in the mouse is a well-established model [44,45]. Recent studies have found that the administration of a chronic lipophilic iron diet for 12 months significantly elevated brain iron and L-ferritin stores in C57BL6 mice and APP mice, respectively [46,47]. This present study investigated whether chronic administration of high concentrations of iron could alter brain iron levels, and then contribute to neuronal degeneration or the formation of Aβ plaques in vivo. In contrast to the aforementioned studies, but in accordance with our previous study using *APP/PS1* mice [48], exposure to chronic high iron in drinking water resulted in a nonsignificant elevation (*p* > 0.05) in brain iron levels in both WT and *APP/PS1* mice; however, compared with the control mice, the HDI-treated mice showed upregulated expression of DMT1 and Fpn, and downregulated expression of TFR. Apparently, neurocytes can both inhibit ferric iron internalization into the cytosol via downregulating TFR, so that the internalized ferric iron is reduced to ferrous iron via upregulating DMT1, and encourage ferrous iron export by upregulating Fpn [7,49]. This suggests that neural cells may be protected by an increased rate of iron export under such conditions. In addition, Perl’s staining analysis revealed no significant difference in iron-positive cells in the cortex and hippocampal regions between WT and *APP/PS1* mice. Aβ has been shown to bind ferrous ions [50], suggesting that accumulation of Aβ might sequester iron and contribute to iron homeostasis in the brain of *APP/PS1* mice. These results suggest that the brain has mechanisms to tightly control the level of iron entering the brain and neurocytes, but these mechanisms may become corrupt with age and/or chronic administration [51,52].

Our previous work revealed that chronic iron exposure increases Aβ deposition, tau hyperphosphorylation, and synapse loss, leading to an exacerbation of cognitive dysfunction in 9-month-old *APP/PS1* mice [48,53,54]. Although the dose of iron used in this experiment was much lower than that used in the previous work, our iron-loaded mouse models showed an apparent increase in Aβ plaque in 12-month-old *APP/PS1* mice, and a possible decrease in NeuN expression in 12-month-old WT and *APP/PS1* mice, when compared to the respective controls. Using Western blot, we confirmed that the expression level of NeuN protein was significantly decreased by HDI treatment. Meanwhile, Nissl body levels were also decreased by HDI treatment in both *APP/PS1* mice and WT littermate controls, indicating that the lower level of NeuN expression could be attributable to iron-induced neuron loss. However, iron overload in our model also impacted the gliocyte population, as we observed a significant increase in GFAP-positive astrocytes and Iba1-positive microglia. Our data are consistent with reports that iron accumulation and overload are associated with an increase in number, and greater activation of, microglia and astrocytes in aged brains, as well as in brains from individuals who had AD [46,55,56,57,58,59]. Astrocytes are specialized for blockading and detoxifying nontransferrin-bound iron [60,61], and microglia show exponentially higher iron content than neurons or astrocytes [62]. Therefore, astrocytes and microglia may play a crucial role in maintaining neuronal function in the HDI-treated brain, through iron trafficking and storage [63].

Next, we aimed to understand the mechanisms underlying the increase in neuron loss observed in HDI-treated WT and *APP/PS1* mouse brains. A variety of neurodegenerative diseases are associated with iron deposition and accompanied by brain tissue oxidative stress injury [64]. ROS levels reflect intracellular oxidative stress, and MDA content indirectly reflects the severity of the free radical attack on cells. As expected, the results showed that the levels of ROS and MDA were significantly increased after HDI treatment, suggesting that HDI induced and aggravated oxidative stress in both the WT and *APP/PS1* mouse brains, especially in the former. Concurrently, a decrease in the expression of the antioxidant enzymes xCT, GPX4, and SOD1 was associated with increased free radical generation in the *APP/PS1* mouse brain after HDI treatment, whereas the expression levels of these antioxidants were significantly increased by HDI treatment in the WT mouse brain. These observations indicate that the iron alterations not only induce oxidative damage, but also trigger sufficient cellular compensation under normal physiological conditions [65], while the compensatory response to iron-induced oxidative damage may be restricted under conditions of Aβ accumulation. Although the specific molecular targets by which iron induces oxidative stress are not known, iron is known to be involved in brain oxidative mitochondria dysfunction and DNA damage [65,66]. Here, our results showed that high-iron treatment significantly reduced the expression of three DNA repair proteins (MUTYH, OGG1 and MTH1) in the brain, suggesting that high-iron treatment decreased the rate of DNA repair and increased DNA oxidation and damage, finally causing the downstream events leading to cell death [67,68].

In fact, iron and ROS have been accepted as important mediators of cell death in many pathological processes that involve altered iron homeostasis. Studies have shown that iron overload in the AD brain can induce oxidative stress, leading to protein aggregation and neuron vulnerability [69,70]. Thus, we further determined which phenotype of neuron death is induced by long-term iron exposure in our system. Caspase is a major participant in AD-related apoptotic cascades, and AIF-induced cell death may also contribute to the neuron death observed in chronic neurodegenerative diseases [71]. As expected, our results showed that the expression of the apoptosis-promoting proteins Bax, PARP1, and AIF was increased after caspase 3 was cleaved in the brain of HDI-treated WT and *APP/PS1* mice. Although the ratio of Bcl-2/Bax was not significantly reduced by HDI treatment, we can still speculate that caspase-dependent apoptosis took place in our system. We then examined levels of autophagy markers (m-TOR, calpain-1, Beclin-1, LC3A/B, and p62) in 12-month-old WT and APP/PS1 mice with or without chronic iron exposure. We found that levels of p-m-TOR were dramatically decreased by HDI treatment, whereas the levels of calpain-1, Beclin-1, LC3A/B, and p62 were elevated. m-TOR is a variety of intracellular protein receptor that regulates the occurrence of autophagy [39]. The observed suppressed phosphorylation of m-TOR indicated that autophagy is induced by HDI treatment in the brain of both WT and APP/PS1 mice [72]. Calpain, which acts independently of m-TOR [40], can participate in intracellular autophagy and apoptosis by cleaving various protein substrates, which is a pathway that is known to be negatively regulated by m-TOR [73]. Interestingly, our previous data provided a possible link between LA and the decreased calcium content of brain tissue in LA-treated P301S Tau transgenic mice [19]. Combined with our current data, these observations suggest that HDI treatment leads to calcium-dependent calpain 1 activation, a decrease in the number of mitochondria, and subsequent activation of autophagy and apoptosis [73,74]. Furthermore, the protein expression levels of Beclin-1, LC3A/B, and p62, several reliable markers of autophagy [41], were significantly higher in HDI-treated mice than those in the respective controls. Despite conflicting knowledge about the role of autophagy in AD [75], these results indicate that autophagy was induced or elevated by HDI treatment, and may augment the neuronal loss triggered by chronic iron exposure. In addition to the aforementioned “classic” mechanisms of neuron loss, an iron- and lipid-peroxidation-dependent pathway of cell death, called ferroptosis, is currently being investigated as a possible pathomechanism in AD [24,76,77]. Here, we found that under normal physiological conditions, chronic iron exposure can not only increase the production of ROS, but also activate the antioxidant enzymes xCT and GPX4, which inhibit the occurrence of ferroptosis [78]. However, long-term high-iron treatment may increase ROS but decrease the levels of xCT and GPX4, suggesting that ferroptosis is induced or increased in the brain tissues of iron-treated *APP/PS1* mice. Collectively, these in vivo studies clearly indicate that chronic iron exposure results in neuronal loss due to apoptosis, autophagy, and ferroptosis, hence increasing the risk for the development of AD. Further studies are required to clarify whether ferroptosis is responsible for the iron-mediated cell death observed in the brains of WT mice.

In summary, we demonstrated that chronic high-dose iron exposure interferes with iron homeostasis and distribution, increases oxidative stress, exacerbates mitochondria dysfunction and DNA oxidative damage, and induces neuronal loss (apoptosis, autophagy, and ferroptosis); however, long-term high-dose iron administration following iron overload might trigger protective antioxidant, anti-apoptosis, and autophagy signaling. The present data, together with previous reports [19,48,53,54], suggest that environmental exposure to iron in drinking water or food might contribute to the evolution and progression of AD. Of course, many of our findings are limited to qualitative histological analyses and protein analyses, and the identification of the precise molecular pathways of iron-dependent neuronal loss is required to support our findings.
